# Grade-Level Differences in Teacher Feedback and Students’ Self-Regulated Learning

**DOI:** 10.3389/fpsyg.2020.00783

**Published:** 2020-05-05

**Authors:** Wenjuan Guo

**Affiliations:** Faculty of Education, East China Normal University, Shanghai, China

**Keywords:** teacher feedback, self-regulated learning, grade-level differences, secondary students, Chinese students

## Abstract

This study investigated grade-level differences in teacher feedback, students’ self-regulated learning (SRL), and their relationship. Secondary students participated in the study (*N* = 1,260; 430 10th-, 460 11th-, and 370 12th-graders). Latent factor mean difference analyses suggested that teacher feedback and students’ SRL level varied across grades. Comparatively, 10th-grade teachers were perceived to provide verification feedback, scaffolding feedback, and praise most frequently; 12th-grade teachers were perceived to provide directive feedback and criticism most frequently; and 11th-grade teachers were perceived to provide all types of feedback least frequently. Students’ SRL generally declined as they aged. Results from three-group structural equation modeling indicated that praise generally exhibited the strongest correlations with SRL regardless of grade level; directive feedback was negatively correlated with 10th graders’ SRL but positively correlated with the SRL of 11th and 12th graders; scaffolding and verification feedback were positively correlated with 11th graders’ SRL; and criticism had small correlations with SRL, regardless of grade level.

## Introduction

Self-regulated learning (SRL) is a significant research area in education, and it has garnered attention from educational researchers, educational administrators, and classroom teachers (Lau and Ho, 2016). SRL is defined as learners proactively taking control of their thoughts, feelings, and behaviors to achieve learning goals (Schunk and Zimmerman, 2010). SRL comprises cognitive strategies, metacognitive strategies, and motivation (Pintrich et al., 1991). Examining students’ SRL is an important undertaking since research has consistently indicated that SRL is imperative for students’ academic success and life-long learning (Broadbent and Poon, 2015; Daniel et al., 2016). Facilitating students’ self-regulation should therefore be a main educational objective for schools. As suggested by social cognitive theory, teacher feedback plays a vital role in promoting students’ SRL (Hattie and Timperley, 2007; Pereira et al., 2016; Graham, 2018). Teacher feedback refers to the information provided by the teacher concerning aspects of students’ understanding and performance in learning.

Owing to the grade-level differences in goal structures and instructional practices (Cleary and Chen, 2009), teachers may provide feedback to students in different grades differently (Núñez et al., 2015). For instance, for graduating students in secondary schools, teachers may emphasize performance-goal orientation and provide more grades or marks (Urdan and Midgley, 2003). By contrast, for freshmen, teachers tend to focus on process-goal orientation and provide more scaffoldings or opportunities for dependent learning (Urdan and Midgley, 2003). Furthermore, research has reported a decline in students’ strategy use and motivation as they progress through school (Hong et al., 2009; Yeung et al., 2011; Wigfield et al., 2015; Gaspard et al., 2017).

Notwithstanding the numerous studies on teacher feedback and students’ SRL, very few have investigated whether teachers’ different types of feedback and their relationships with students’ SRL vary across grade levels. In the context of secondary schools, where competition is increasingly keen, students may feel less friendly and have less fun at school as compared to their younger counterparts (Goh and Gopinathan, 2008). In addition, as students age, they may re-evaluate their capabilities and change their self-perceptions (Yeung et al., 2011). These self-reflective processes may cause diverse responses to teachers’ feedback as students progress through higher grades. Many teachers may need to teach students in different grade levels, however, they may know little about whether and how to provide feedback differently to them to promote their learning more effectively.

The present study aimed to bridge the research gap and compare teacher feedback, students’ SRL, and their relationships among different secondary school grade levels. The present study contributes to the field of educational research by adding to the literature on teacher feedback and students’ SRL from the perspective of grade-level differences and offering practical implications for teachers in providing effective feedback for students to cultivate their SRL.

### Teacher Feedback

Research has indicated that teacher feedback is a key factor in improving student learning (Hattie and Timperley, 2007; Perera et al., 2008; Quinton and Smallbone, 2010; Clark, 2012). The main purpose of teacher feedback is to reduce the discrepancies between students’ current understandings and performance and their desired learning goals (Hattie and Timperley, 2007). Teacher feedback can be effective or dysfunctional in promoting student learning, depending on its type (Guo and Wei, 2019).

Researchers have proposed several types of teacher feedback. Since the present study explored the functions of teacher feedback on students’ SRL, this study adopted Guo’s (2017) framework because it was constructed based on the main functions of teacher feedback in student learning and is appropriate for this study. Guo (2017) identified five types of teacher feedback: verification feedback, directive feedback, scaffolding feedback, praise, and criticism. Verification feedback refers to a dichotomous judgment of a student’s response by affirming it as correct or incorrect, such as provided by marks or grades (Black and Wiliam, 1998). Directive feedback refers to teachers telling a student the direct answer to their questions or problems. Scaffolding feedback refers to a series of successive cues, prompts, hints, or partial solutions that consist of breaking down tasks into easier or smaller parts to facilitate students in generating correct answers by themselves (McMillan, 2014). Praise refers to teachers commending the worth of a student’s learning attitudes, behaviors, performance, or products (Brophy, 1981). Lastly, criticism refers to teachers’ negative response to a student’s learning attitudes, behaviors, or performance via expressions of disgust, disapproval, or rejection (Brophy, 1981).

Chinese senior secondary schooling is a 3-year program (grades 10–12) culminating in the national university entrance examination, i.e., *Gao Kao*. *Gao Kao* is very competitive, and all teachers and students must therefore make every endeavor to prepare for it carefully. *Gao Kao* lasts for 2 days, however, almost all students begin their preparation as early as their first day in 10th grade (Guo, 2017). In recent years, around 10 million students each year have registered for *Gao Kao*, with the admission rate of key universities only ranging from 3 to 15% depending on province or city (Intelligent Research Consulting, 2019). For most students, being admitted to a key university is vital to meet their families’ expectations and their ability to obtain satisfactory employment and even earn social status and honor (Zhang, 2013; Tsegay and Ashraf, 2015). Since key senior high schools usually have rich educational resources, they play a significant role in promoting students to pass *Gao Kao* (Liu, 2013). Thus, many students want to attend these senior high schools, and they may take the necessary entrance examinations (i.e., *Zhong Kao*) several times. It is therefore common to find that there is a wide age range per grade.

In the context of *Gao Kao*, teachers’ instructional practices may differ as students progress through higher grades to adapt to students’ unique developmental needs. First, for students in 10th grade, it is significant to master basic subject knowledge and skills and develop good learning habits and abilities to prepare for their future competitive entrance examinations (Urdan and Midgley, 2003; Guo, 2017). To help students achieve this, teachers focus more on mastery- than performance-goal orientation, and they may thus offer more scaffolding feedback to facilitate students in mastering knowledge and more praise to encourage them to develop certain learning habits as desired. Second, for students in the 11th grade, the most important thing remains to master subject knowledge and skills and to consolidate the learning habits that have been developed in the 10th grade. Therefore, teachers may still focus more on mastery-goal orientation but may provide less feedback than in the 10th grade since the 11th grade is a transitional phase–from the first year of high school to the graduating year (Guo, 2017). Third, for students in the 12th grade, their main learning goal is to practice and revise, testing the material taught intensively in the previous 2 years to meet the upcoming *Gao Kao*. Teachers in this grade transform from mastery- to performance-goal orientation, and their main instructional goal is to facilitate students to increase test scores (Cleary and Chen, 2009; Hong et al., 2009). Therefore, their feedback may be more summative as compared to teachers in the other grade levels.

### SRL

Given the significant role of SRL in students’ academic achievement and continuous learning in and beyond school, SRL is a key element in many countries’ educational systems (Daniel et al., 2016; Randi, 2017; Zhu and Mok, 2018). Despite the diversity of existing SRL models, there is a consensus that SRL is a constructive process in which students proactively manage their cognitive, metacognitive, and motivational processes (Panadero, 2017). Cognitive strategies include four components: (1) rehearsal, naming, or reciting items from a list to be learned; (2) elaboration, the capability to build internal connections between to-be-learned items; (3) organization, the ability to select information appropriately and make connections among different pieces of information; and (4) critical thinking, the ability to solve problems and make evaluations or decisions critically according to certain standards (Pintrich et al., 1991). Metacognitive strategies comprise metacognitive awareness, which refers to awareness and reflection on one’s own cognition, and metacognitive monitoring and management, which means planning, monitoring, and management one’s own cognition (Flavell, 1979; Pintrich et al., 1991). Motivation involves four dimensions: (1) intrinsic motivation, the degree that learners take in a task owing to their curiosity, mastery, or challenge; (2) extrinsic motivation, the degree to which students take in a task to obtain greater performance, higher grades, external reward, or competition with others; (3) self-efficacy, the degree to which students perceive their ability to accomplish a task successfully; and (4) test anxiety, a series of anxieties before a test, such as negative affect, thoughts, and psychological arousal (Pintrich et al., 1991).

Researchers have suggested that self-regulated learners are more likely to proactively and skillfully choose and adapt effective cognitive strategies to better address learning problems and challenges (Schunk and Zimmerman, 2010). In addition, self-regulated learners are aware of their cognitive status and know clearly how to apply different strategies to monitor, control, and regulate their cognition (Flavell, 1979). Self-regulated learners are also highly motivated and self-confident in what they are learning, and they are also good at controlling their emotions (Pintrich et al., 1991). Suggested by de la Fuente, SRL may result from the combined effects of self-regulation and externally regulated learning (ERL; de la Fuente et al., 2017; de la Fuente-Arias, 2017). The learning context can promote students’ positive or adequate proactivity and facilitate their SRL. For instance, teachers can externally regulate the learning process by offering necessary stimuli and aids to foster students’ SRL (de la Fuente-Arias, 2017).

While SRL is well known to influence student learning, most previous studies have generally reported a decline in students’ SRL, i.e., various strategies used and motivation, when they progress through higher grades (Cleary and Chen, 2009; Hong et al., 2009; Lau, 2009; Yeung et al., 2011; Huang, 2013; Wigfield et al., 2015; Gaspard et al., 2017). For instance, research has indicated that students in higher grade levels generally reported poorer motivation than did students in lower grade levels (Wigfield et al., 2015; Gaspard et al., 2017). Researchers have also found that students in the seventh grade exhibited a less adaptive regulatory and motivation profile than did their younger peers (Cleary and Chen, 2009). In addition, research indicated that students exhibited a significant decline in their homework self-regulation when they reached higher grades (Huang, 2013).

### Relationships Between Teacher Feedback and Students’ SRL

As suggested by social cognitive theory (Bandura, 2011), students’ SRL can be influenced by the social environment. Teacher feedback, serving as one of social environmental factor, can exert a strong influence on students’ SRL (Pereira et al., 2016). Research has indicated that scaffolding feedback has a strong influence on students’ self-regulated strategy use and motivation by facilitating students to be independent gradually via hints, prompts, or clues (Finn and Metcalfe, 2010; McMillan, 2014; Guo, 2017; Guo and Wei, 2019; Hyland and Hyland, 2019). For instance, Guo and Wei (2019) found that scaffolding feedback could effectively promote students’ use of metacognitive strategies, resource management strategies, intrinsic motivation, and self-efficacy. In addition, research has indicated that praise, which is often used in the West to motivate students, may have a negative effect on students’ perceptions of their abilities (Salili and Hau, 1994; Hattie and Timperley, 2007; Skipper and Douglas, 2012). In contrast, Chinese teachers and parents always hold high expectations for children and seldom praise them; they consider praise as having the ability to spoil them if given without an outstanding cause (Chao, 1994; Salili and Hau, 1994; Guo, 2017). Chinese students tend to perceive that praise is associated only with greater ability, diligence, or a host of other virtues. Thus, teachers’ praise after success may promote students’ SRL.

However, teacher feedback can also be ineffective or even detrimental to students’ development of SRL. Research has indicated that verification feedback, such as grades or marks, may decrease students’ intrinsic motivation and self-efficacy by distracting their focus from accomplishing tasks to competing with peers (Lipnevich and Smith, 2008). Directive feedback was also found to have detrimental effects on students’ SRL. When teachers directly tell students correct answers or full solutions of problems, students may increasingly become dependent on teachers and reluctant to solve difficult problems or meet challenges independently (Shute, 2008; Guo and Wei, 2019). However, research has suggested that if students perceive such teacher-led feedback as positive, its negative effects on student learning may be lessened (Harris et al., 2014). Furthermore, teachers’ criticism may decrease students’ intrinsic motivation, self-efficacy, and use of various strategies in learning (Atlas et al., 2004; Guo, 2017). However, in Chinese collectivistic contexts, research has indicated that the value placed on shame may emerge at the same time students are learning to adjust to group norms, namely, they may be helped when shamed (Wong and Tsai, 2007). This suggests that criticism that shames students may play a positive role in Chinese students’ SRL.

Therefore, based on the possible effects of teacher feedback on student learning and de la Fuente-Arias’s (2017) externally regulated learning (ERL) theory, I categorize the five types of feedback into (1) external regulator (i.e., scaffolding feedback and praise), referring to the positive external factors that can promote students’ SRL; (2) a-regulator (i.e., verification and directive feedback), referring to the external factors that may put students at the mercy of the external regulatory system of the context; and (3) de-regulator (i.e., criticism), referring to the external factors that may impede students’ SRL.

As students progress through higher grades, the influence of teacher feedback on their SRL may differ from that in lower grades. First, as students age, they may re-evaluate their competencies based on teachers’ feedback and become more realistic rather than overly optimistic or exaggerated (Lau, 2009). This may result in students adapting their responses to teacher feedback. For instance, they may not feel too proud when praised by teachers or too pessimistic or sad when criticized (Guo, 2017). Some social factors, such as a better economic situation, may compensate for such effects of age on self-evaluation (Dunning et al., 2004). Second, the school environment or climate in higher grades may become more impersonal, more formal, and more evaluative, which may moderate the effects of teacher feedback on students’ SRL (Lau, 2009). The heavy use of extrinsic incentives in higher grades may also lead to the progressive undermining of students’ intrinsic motivation and self-efficacy in learning difficult subjects, even with teachers’ positive feedback. Finally, the change of students’ learning goals from mastery to performance may also play a role. Students may focus more on competition with peers, such as entrance examinations, when they are in higher (vs. lower) grades (Hong et al., 2009). This may cause them to place a high utility value on learning and become more anxious about their performance ranking in school. Therefore, they may be more negatively affected by teachers’ criticism or lower marks or grades as compared to their peers.

Despite the large body of research on teacher feedback and SRL, some research gaps exist in the literature. First, most previous studies examined teacher feedback in general (e.g., Hattie and Timperley, 2007; Perera et al., 2008; McMillan, 2014; Guo and Wei, 2019) or the differences between male and female teachers in providing feedback (e.g., Centra and Gaubatz, 2000; MacNell et al., 2015). Very few researchers have examined whether teachers provide feedback to students in different grades differently. Knowing this can elucidate teachers’ instructional practices and tactics across grades from the perspective of feedback.

Second, although many studies have indicated a decline in students’ SRL as they age, most were conducted in Western countries. Few related studies were based in Chinese cultural settings, where the school system is highly examination driven (Hong et al., 2009). Thus, it is necessary to iterate the existing research in the Chinese secondary school context.

Finally, of the limited studies on the relationship between teacher feedback and students’ SRL (e.g., Guo, 2017; Guo and Wei, 2019), very few studies have examined the grade differences in such relationships. Many teachers may need to teach students in different grades; however, they may know little about whether and how they should provide diverse feedback for students in different grades to effectively promote their learning. Therefore, it is necessary to explore whether and how different types of teacher feedback may affect different grade students’ SRL and in what pattern. Consequently, early instructional interventions can be implemented to effectively promote different grade students’ SRL.

### Research Questions and Hypotheses

This study aimed to bridge these noted gaps by exploring the grade-level differences in teacher feedback, students’ SRL, and their relationship patterns in the context of Chinese secondary schools. Specifically, I addressed several research questions (RQs) and hypotheses (H):

RQ1. Are there any differences in teachers’ feedback to students in different grades?

H1. In the Chinese context of *Gao Kao*, the main learning goals of students in different grade levels differ (e.g., Yeung et al., 2011; Guo, 2017). To help students in different grade levels learn effectively, teachers may adapt their instructional practices such as feedback (Cleary and Chen, 2009; Hong et al., 2009). Thus, I expect that teachers’ feedback to students in different grade levels may vary.

RQ2. Are there grade-level differences in Chinese students’ SRL?

H2. Based on the previous research findings that students in higher grades had lower levels of strategy use and motivation than did those in lower grades owing to changes in their self-perceptions and the school environment/climate (Cleary and Chen, 2009; Hong et al., 2009; Lau, 2009; Yeung et al., 2011; Huang, 2013), I expect that Chinese students in this study will also report a general decline in the various components of SRL.

RQ3. Are there grade-level differences in the relationships between different types of teacher feedback and students’ SRL?

H3. Since there is a change in the school environment/climate and students’ self-perceptions and learning goals as they progress through higher grades, students may change their attitudes and responses to teachers’ feedback (Hong et al., 2009; Lau, 2009; Guo, 2017). Therefore, I hypothesized that the relationship between teacher feedback and students’ SRL may also differ across grades.

## Materials and Methods

### Participants

Participants were made up of 1,332 secondary students in Shanghai, China. All voluntarily completed the questionnaires, however, 1,260 (95%) were valid: (430; 34%) 10th graders, (460; 37%) 11th graders, and (370; 29%) 12th graders). The 10th graders were aged 15 to 18 years, with an overall mean age of 15.90 years (*SD* = 0.60); the 11th graders were aged 15 to 19 years, with an overall mean age of 16.78 years (*SD* = 0.61); and the 12th graders were aged 17 to 20 years, with an overall mean age of 17.64 years (*SD* = 0.67). Table 1 provides more information about the participants. The participating school is in an urban area and has adequate advanced learning resources including high-tech learning devices. This school has a similar curriculum to other schools in China, and Mandarin Chinese is a compulsory subject and is also the language of instruction. However, in addition, this school also has several school-based curricula (e.g., a piano course, art course, and football course), which may differ from other schools. Students at this school mainly come from Shanghai, and a few come from other cities or areas of China. The teacher/student ratio is approximately 1:16. Most teachers teach the same students when they reach the higher grade levels, and a few may teach in one grade level for several years.

**TABLE 1:** Demographics of the participating students in this study.

	10th graders	11th graders	12th graders
Number of participants	430 (34%)	460 (37%)	370 (29%)
Average age	15.90 (*SD* = 0.60)	16.78 (*SD* = 0.61)	17.64 (*SD* = 0.67)
**Gender**			
Number of Males	180 (42%)	212 (46%)	154 (42%)
Number of Females	250 (58%)	248 (54%)	216 (58%)

### Measures

#### Teacher Feedback Measures

The teacher feedback measures developed by Guo (2017) were used to ask students to report their teachers’ use of different types of feedback. Teachers’ feedback can be oral or written. This questionnaire included five scales: verification feedback, directive feedback, scaffolding feedback, teacher praise, and criticism. All items were rated on a six-point Likert scale (1 = *my teacher never does this* to 6 = *my teacher always does this*). The mean of items for each subscale was calculated separately, with higher numbers indicating that type of feedback was more frequently provided by the teacher. Sample items, descriptive statistics, and internal consistencies for all the scales are shown in Table 2. The reliability of all subscales ranged from 0.75 to 0.88, suggesting that the items of each feedback scale had acceptable internal consistency.

**TABLE 2:** Items, internal consistency coefficients, and descriptive statistics for the variables of teacher feedback measured in this study.

	Cronbach’s α	Sample item	M (SD)
Verification feedback	0.75	(1) My teacher gives a score or grade on our quiz.	5.19 (1.29)
		(2) My teacher indicates whether my answers in the class are correct.	5.57 (0.81)
		(3) My teacher points out where I am right and wrong in my homework.	5.45 (0.95)
		(4) My teacher points out the places that are not completely correct or incomplete in our homework.	5.34 (1.04)
		(5) My teacher tells me what knowledge points I have mastered.	4.80 (1.35)
Directive feedback	0.80	(6) My teacher directly tells me the correct answer when I get an answer wrong in the classroom.	4.46 (1.43)
		(7) When I give an incomplete answer, my teacher directly supplements it for me.	4.46 (1.43)
		(8) When I make mistakes in my homework, my teacher directly corrects it for me.	5.32 (1.03)
		(9) My teacher directly tells me how to solve difficult problems in my homework.	5.02 (1.19)
		(10) My teacher directly tells us the correct answers to the test questions.	4.45 (1.45)
Scaffolding feedback	0.81	(11) My teacher helps me solve problems by offering some hints or cues.	5.20 (1.09)
		(12) My teacher helps us to complete difficult problems by breaking down the problem or reducing the difficulty.	4.93 (1.15)
		(13) To help us do our homework, my teacher reminds us of some learning methods or techniques.	5.23 (1.01)
		(14) To help us do our homework, my teacher reminds us of the task requirements or evaluation criteria.	5.06 (1.12)
Teacher praise	0.88	(15) When my academic performance is better than that of other students, my teacher praises me.	4.89 (1.26)
		(16) My teacher praises me when I perform better than before.	4.89 (1.26)
		(17) When I get a correct answer to difficult questions in the class, my teacher praises me.	4.48 (1.38)
		(18) When I get a correct answer to difficult questions in tests, my teacher praises me.	4.58 (1.39)
		(19) When my ranking in the exam progresses, my teacher praises me.	4.47 (1.38)
Teacher criticism	0.86	(20) My teacher criticizes or punishes me when I fail an exam.	4.27 (1.37)
		(21) When my performance is not as good as before, my teacher criticizes me.	3.98 (1.50)
		(22) When my academic performance is worse than that of other students, my teacher criticizes me.	4.17 (1.45)
		(23) When my ranking in the exam goes backward, my teacher criticizes me.	3.99 (1.50)
		(24) When our class does worse on a test as compared to another class, my teacher criticizes us.	3.68 (1.65)

#### Motivated Strategies for Learning Questionnaire (MSLQ)

Items to measure students’ SRL in this study were adopted from the MSLQ, which was developed by Pintrich et al. (1991). All items were measured on a seven-point Likert scale (1 = *I strongly disagree* to 7 = *I strongly agree*). This questionnaire was translated into Chinese by the author based on the Chinese classroom context, and then backtranslated by a professor fluent in both Chinese and English.

To ensure its content validity, four professors in this research area, two front-line Chinese-language teachers, and three doctorate students were invited to review the items. These items were first reviewed by each professor and doctorate student to examine the content structure. After a few rounds of revisions, formal interviews with the two Chinese-language teachers from the participating school were conducted to ensure that the SRL measurements appropriately captured students’ SRL. All finalized items were jointly determined by the researchers and independent advisers. Three scales comprising 10 subscales are included in the questionnaire. The mean of items for each subscale was separately taken, with higher numbers indicating more use of strategies or greater motivation for learning. Sample items, descriptives, and internal consistencies for the subscales are shown in Table 3. The reliability of all subscales ranged from 0.76 to 0.91, suggesting that the items of each SRL scale also had acceptable internal consistency.

**TABLE 3:** Sample items, internal consistency coefficients, and descriptive statistics for the variables of students’ self-regulated learning measured in this study.

Scales	Cronbach’s α	Sample item (no. of items in the scale)	M (SD)
**Cognitive strategies**			
Rehearsal	0.81	When I study for this class, I practice saying the material to myself over and over (5)	4.75 (1.08)
Elaboration	0.80	When reading for this class, I try to relate the material to what I already know (5)	4.48 (1.15)
Organization	0.77	I make simple charts, diagrams, or tables to help me organize course material (5)	3.94 (1.27)
Critical thinking	0.87	I treat the course material as a starting point and try to develop my own ideas about it (5)	3.94 (1.25)
**Metacognitive strategies**			
Metacognitive awareness	0.82	I know it is necessary to set a plan and steps for my learning (5)	4.85 (1.02)
Metacognitive monitoring and management	0.83	I will modify my learning methods for adjusting to learning materials and teachers’ instruction (5)	4.49 (1.08)
**Motivation**			
Intrinsic motivation	0.88	I prefer course material that arouses my curiosity, even if it is difficult to learn (5)	4.77 (1.17)
Extrinsic motivation	0.88	Getting a good grade in this class is the most satisfying thing for me right now (5)	5.19 (1.18)
Self-efficacy	0.88	I’m certain I can master the skills being taught in this class (5)	4.62 (1.19)
Test anxiety	0.91	I feel my heart beating fast when I take an exam (5)	4.55 (1.54)

### Procedures

All participants volunteered to complete the questionnaires. Permission was obtained from the school’s principals and teachers, and written informed consent was obtained from all participants. Before the questionnaires were administered, the author read aloud a brief set of directions concerning how to complete the questionnaires and provided some practice questions. All participants were informed that the data collected would be kept confidential and used only for research purposes. The two questionnaires took approximately 18 min to complete, and all questionnaires were collected immediately after their completion during a mandarin course.

### Data Analyses

I conducted four sets of analyses. First, to evaluate the equivalence of teacher feedback and SRL measures for students in each grade, I conducted a series of three-group confirmatory factor analyses (CFAs) using Mplus 7 (Muthén and Muthén, 1998–2010) to examine the configural, metric, scalar, and residual invariance of the measures for the three grades separately. An unconstrained model was compared with a constrained model for each measure. Second, I calculated CFAs using Mplus 7 to examine the overall factor structure of the two measures. Third, I computed latent factor mean difference analyses using Mplus 7 to examine any grade-level differences in teacher feedback and students’ SRL separately. Finally, I conducted three-group structural equation modeling (SEM) using Mplus 7 to examine any grade-level differences in the correlations between teacher feedback and students’ SRL.

## Results

### CFAs

**FIGURE 1:**
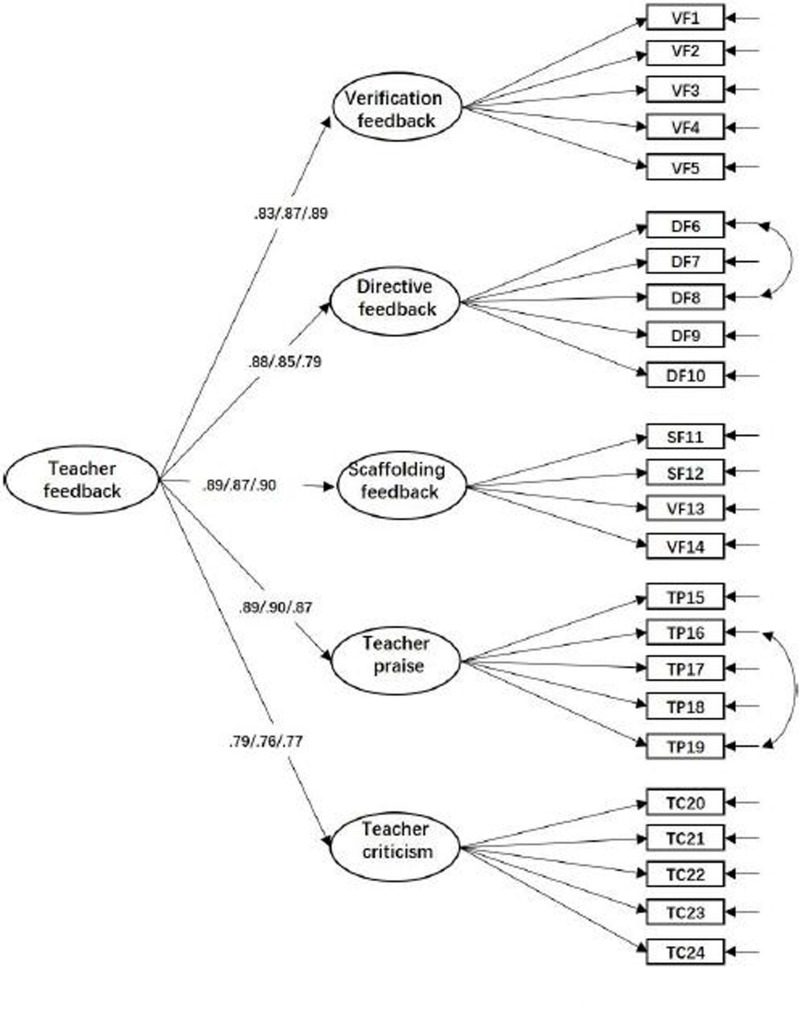
Confirmatory factor analysis of teacher feedback measures.

**FIGURE 2:**
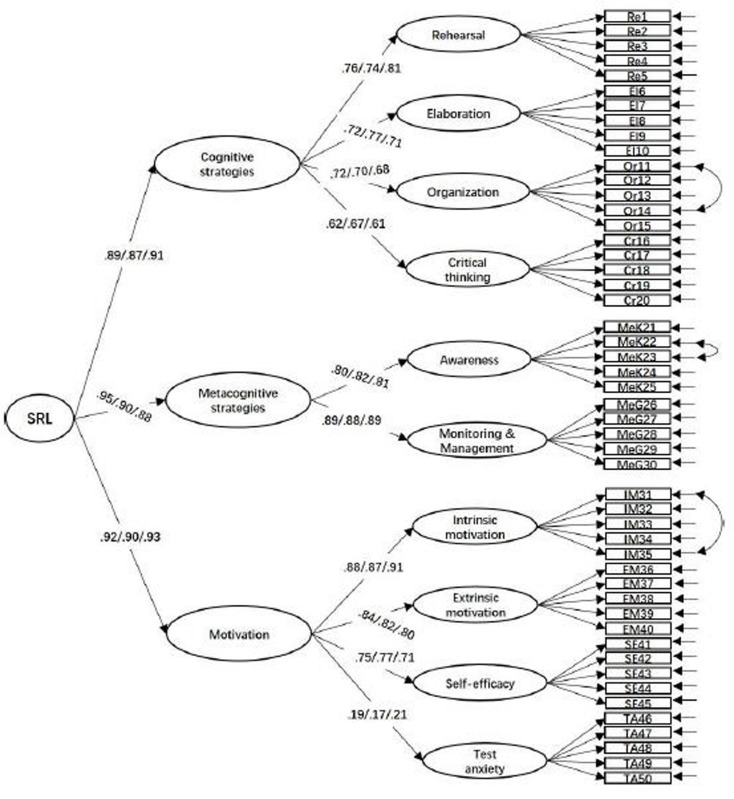
Confirmatory factor analysis of SRL measures.

Two CFAs were conducted separately to examine the factor structure of the items measuring teacher feedback and students’ SRL. In addition, three-group CFA were conducted for feedback and SRL measures to examine whether the factor structure of feedback and SRL were valid for 10th-, 11th-, and 12th-grade students. In terms of feedback measures, as shown in Figure 1, the measurement model fit the data well for all three grades, χ^2^ = 1,205.184; *df* = 715; *p* < 0.001; RMSEA = 0.040; 90% CI [0.036,0.044], CFI = 0.915; TLI = 0.906; SRMR = 0.070. All the factor loadings and correlations reached significance (βs = 0.76 to 0.90, *r*s = 0.33 to 0.56, *p*s < 0.05). In terms of SRL measures, as shown in Figure 2, the measurement model fit the data well for all three grades, χ^2^ = 3,567.243; *df* = 2,703; *p* < 0.001; RMSEA = 0.033; 90% CI [0.030,0.036], CFI = 0.919; TLI = 0.903; SRMR = 0.058. All the factor loadings and correlations reached significance (βs = 0.17 to 0.91, *r*s = 0.42 to 0.75, *p*s < 0.05). Though some residuals of the two measures were correlated, there were no significant changes in the estimation of the factor loadings, and these thus may not inflate the fit quality of the CFAs (Bagozzi, 1983).

### Measurement Invariance

To ensure that the teacher feedback measures and SRL measures were equivalent for students across grades, a series of nested CFAs were computed to examine the metric and scalar invariance of the measures. Metric invariance permits comparisons of the associations, while scalar invariance permits comparisons of the means. First, a fully unconstrained three-group CFA model, in which parameters were freely estimated, was conducted to examine the measurement structure of latent factors; this was treated as the baseline model for further analysis. As shown in Tables 4, 5, all the unconstrained models fit the data adequately [χ*^2^*(*df*s = 8–376)s < 632.410, CFIs > 0.928, TLIs > 0.921, RMSEAs < 0.063]. Second, a constrained three-group CFA model was computed in which the factors and factor loading patterns were constrained to be equal across three groups. All the constrained models for metric equivalence fit the data adequately [χ*^2^*(*df*s = 10–421)s < 712.356, CFIs > 0.924, TLIs > 0.918, RMSEAs < 0.061]. All the constrained models for scalar equivalence also fit the data adequately [χ*^2^*(*df*s = 13–453)s < 766.452, CFIs > 0.923, TLIs > 0.916, RMSEAs < 0.063]. Third, the changes in CFIs and RMSEAs from the unconstrained to metric models, and from metric to scalar models were less than 0.01, indicating that all the teacher feedback and SRL measures met the criteria for both metric and scalar invariance (Cheung and Rensvold, 2002; Chen, 2007; see Tables 4, 5).

**TABLE 4:** Measurement invariance tests for the teacher feedback measures.

Scales	Model and invariance level	Over fit indexes					Model comparison	Comparative fit indexes	
		χ^2^	*df*	CFI	TLI	RMSEA		ΔCFI	ΔRMSEA
Verification feedback	(1) Unconstrained	9.432	8	0.998	0.994	0.029			
	(2) Metric	11.651	10	0.997	0.990	0.028	2 vs. 1	0.001	0.001
	(3) Scalar	14.213	13	0.997	0.987	0.031	3 vs. 2	0.000	0.003
Directive feedback	(1) Unconstrained	25.479	20	0.977	0.968	0.061			
	(2) Metric	38.663	22	0.973	0.963	0.060	2 vs. 1	0.004	0.001
	(3) Scalar	53.756	25	0.968	0.960	0.063	3 vs. 2	0.005	0.003
Scaffolding feedback	(1) Unconstrained	17.465	14	0.995	0.994	0.036			
	(2) Metric	21.845	18	0.992	0.992	0.032	2 vs. 1	0.003	0.004
	(3) Scalar	40.578	21	0.990	0.991	0.036	3 vs. 2	0.002	0.004
Teacher praise	(1) Unconstrained	30.764	16	0.991	0.988	0.061			
	(2) Metric	36.334	22	0.987	0.983	0.056	2 vs. 1	0.004	0.005
	(3) Scalar	42.547	29	0.984	0.980	0.060	3 vs. 2	0.003	0.004
Teacher criticism	(1) Unconstrained	45.265	25	0.980	0.981	0.063			
	(2) Metric	55.002	31	0.979	0.980	0.061	2 vs. 1	0.001	0.002
	(3) Scalar	65.901	42	0.978	0.976	0.063	3 vs. 2	0.001	0.002

*In the unconstrained models the parameters were freely estimated, whereas in the constrained models the factor loadings and intercepts for each item were forced to be equal across grades. χ^2^, Chi-square statistic; RMSEA, robust root-mean-square error of approximation; CFI, robust comparative fit index; TLI, Tucker Lewis index; Δ, difference between the comparison and nested model for each scale.*

**TABLE 5:** Measurement invariance tests for the SRL measures.

Scales	Model and invariance level	Over fit indexes					Model comparison	Comparative fit indexes	
		χ^2^	*df*	CFI	TLI	RMSEA		ΔCFI	ΔRMSEA
Cognitive strategies	(1) Unconstrained	567.584	265	0.949	0.923	0.060			
	(2) Metric	652.685	309	0.943	0.921	0.058	2 vs. 1	0.006	0.002
	(3) Scalar	742.674	351	0.942	0.920	0.060	3 vs. 2	0.001	0.002
Metacognitive strategies	(1) Unconstrained	465.212	124	0.932	0.935	0.059			
	(2) Metric	519.706	141	0.925	0.933	0.053	2 vs. 1	0.007	0.006
	(3) Scalar	574.500	171	0.923	0.932	0.054	3 vs. 2	0.002	0.001
Motivation	(1) Unconstrained	632.410	376	0.928	0.921	0.059			
	(2) Metric	712.356	421	0.924	0.918	0.057	2 vs. 1	0.004	0.002
	(3) Scalar	766.462	453	0.923	0.916	0.060	3 vs. 2	0.001	0.003

*In the unconstrained models the parameters were freely estimated, whereas in the constrained models the factor loadings and intercepts for each item were forced to be equal across grades. χ2, Chi-square statistic; RMSEA, robust root-mean-square error of approximation; CFI, robust comparative fit index; TLI, Tucker Lewis index; Δ, difference between the comparison and nested model for each scale.*

### Grade-Level Differences in Teacher Feedback

As demonstrated in Table 6, the results of latent factor mean difference analyses indicated that, for verification feedback (VF) and directive feedback (DF), 10th- (*M*_VF_ = 5.42; *M*_DF_ = 4.88) and 12th-grade teachers (*M*_VF_ = 5.27; *M*_DF_ = 4.89) were reported to provide more feedback than did 11th-grade teachers (*M*_VF_ = 5.09; *M*_DF_ = 4.53; *p*s < 0.001), however, no significant differences were found between 10th- and 12th-grade teachers (*ps* > 0.05). Concerning scaffolding feedback, 10th-grade teachers (*M* = 5.31) were reported to provide more than did 11th- (*M* = 4.95) and 12th-grade teachers (*M* = 5.06; *p*s < 0.001), however, no significant differences were found between 11th- and 12th-grade teachers (*p* > 0.05). In addition, 10th- (*M* = 4.98) and 12th-grade teachers (*M* = 4.16) were reported to provide the most praise and criticism, and 11th-grade teachers were reported to provide the least praise (*M* = 4.43) and criticism (*M* = 3.87; *p*s > 0.05). However, as shown in Table 5, despite the statistical significance, most of the effect sizes (i.e., standardized mean differences) were under 0.40 and small (Hattie, 2009). Therefore, overall, the grade-level differences in teacher feedback were trivial to small.

**TABLE 6:** Grade-level differences of teacher feedback.

Variables	G10 vs. G11	G10 vs. G12	G11 vs. G12
Verification feedback	−0.324*** (−0.099)	−0.163(−0.035)	0.218***(0.096)
Directive feedback	−0.370*** (−0.182)	0.020(0.007)	0.390***(0.204)
Scaffolding feedback	−0.439*** (−0.310)	−0.330***(−0.214)	0.144(0.106)
Teacher praise	−0.544*** (−0.502)	−0.378***(−0.334)	0.178*(0.164)
Teacher criticism	−0.176*** (−0.173)	0.088***(0.094)	0.306***(0.283)

a, * p < 0.05, ** p < 0.01, *** p < 0.001. b, the results are derived from latent mean structures analyses, in which 10th graders were coded as the reference group (mean = 0) in the left and middle columns, and 11th graders were coded as the reference group in the right columns. Thus, the negative numbers reflect that the higher grade is lower than the lower grade (i.e., the reference group). c, standardized means (11th graders in the left column and 12th graders in the middle and right columns) are on the left of parentheses, and unstandardized means are in parentheses. d, the SEM models fit the data adequately, χ^2^s > 21.125, CFIs > 0.915, TLIs > 0.921, RMSEAs < 0.079.

### Grade-Level Differences in Students’ SRL

As shown in Table 7, the latent factor mean difference analyses suggested that concerning the use of rehearsal (Re) and elaboration strategies (EI), 11th-grade students (*M*_Re_ = 4.83; *M*_EI_ = 4.58) reported higher levels than did 12th-grade students (*M*_Re_ = 4.63; *M*_EI_ = 4.39), however, no significant differences were found between 10th- and 11th-grade students nor 10th- and 12th-grade students (*p*s > 0.05). Concerning the use of organization (Or) strategies, metacognitive monitoring and management (MMM) strategies, and intrinsic motivation (IM), both 10th- (*M*_Or_ = 3.99; *M*_MMM_ = 4.49; *M*_IM_ = 4.89) and 11th-grade students (*M*_Or_ = 4.11; *M*_MMM_ = 4.61; *M*_IM_ = 4.78) reported higher levels than did 12th-grade students (*M*_Or_ = 3.68; *M*_MMM_ = 4.33; *M*_IM_ = 4.61; *p*s < 0.05), however, no significant differences were found between 10th- and 11th-grade students (*p*s > 0.05). Concerning extrinsic motivation, 10th-grade students (*M* = 5.39) reported a higher level than did 11th- (*M* = 5.03) and 12th-grade students (*M* = 5.15; *p*s < 0.05), however, no significant difference was found between 11th- and 12th-grade students (*p* > 0.05). Whereas, as suggested by the effect sizes in Table 6 (i.e., standardized mean differences), the grade-level differences of SRL were trivial to small.

**TABLE 7:** Grade-level differences of students’ SRL.

Variables	G10 vs. G11	G10 vs. G12	G11 vs. G12
**Cognitive strategies use**			
Rehearsal	−0.011(−0.011)	−0.117(−0.117)	−0.222**(−0.217)
Elaboration	0.110 (0.104)	−0.066(−0.062)	−0.179*(−0.172)
Organization	0.014 (0.015)	−0.272*(−0.317)	−0.384***(−0.437)
Critical thinking	0.007 (0.008)	−0.042(−0.052)	−0.104(−0.119)
**Metacognitive strategies use**			
Awareness	−0.016(−0.013)	−0.075(−0.064)	−0.103(−0.090)
Monitoring and management	0.114 (0.102)	−0.157*(−0.152)	−0.277***(−0.258)
**Motivation**			
Intrinsic motivation	−0.126(−0.125)	−0.304**(−0.339)	−0.151*(−0.155)
Extrinsic motivation	−0.403***(−0.438)	−0.186*(−0.194)	0.130 (0.141)
Self-efficacy	0.000 (0.000)	−0.118(−0.124)	−0.108(−0.118)
Test anxiety	−0.120(−0.167)	0.011 (0.015)	0.109 (0.151)

a, * p < 0.05, ** p < 0.01, *** p < 0.001. b, the results are derived from latent mean structures analyses, in which 10th graders were coded as the reference group (mean = 0) in the left and middle columns, and 11th graders were coded as the reference group in the right columns. Thus, the negative numbers reflect that the higher grade is lower than the lower grade (i.e., the reference group). c, standardized means (11th graders in the left column and 12th graders in the middle and right columns) are on the left of parentheses, and unstandardized means are in parentheses. d, the SEM models fit the data adequately, χ^2^s > 3.435 CFIs > 0.920, TLIs > 0.927, RMSEAs < 0.080.

### Grade-Level Differences in the Relationships Between Teacher Feedback and Students’ SRL

Zero-order correlations were initially computed to explore the relationships between different types of teacher feedback and students’ SRL. As shown in Table 8, verification feedback had significant and positive correlations with students’ SRL, except for the use of organization and critical-thinking strategies. Directive feedback only had significant and positive correlations with students’ use of rehearsal, metacognitive monitoring and management strategies, intrinsic and extrinsic motivation, and test anxiety. Scaffolding feedback had significant and positive correlations with all components of students’ SRL, except for the use of critical-thinking strategies. Teachers’ praise and criticism had significant and positive correlations with all components of students’ SRL.

**TABLE 8:** Zero-order correlations among all variables of teacher feedback and students’ self-regulated learning.

	VF	DF	SF	MP	MC	Re	EI	Or	Cr	MEA	MMM	IM	EM	SE
DF	0.42***													
SF	0.58***	0.40***												
MP	0.42***	0.35***	0.61***											
MC	0.24***	0.26***	0.28***	0.48***										
Re	0.19***	0.11***	0.25***	0.30***	0.13***									
EI	0.09**	0.05	0.17***	0.25***	0.18***	0.57***								
Or	0.04	−0.02	0.15***	0.26***	0.19***	0.46***	0.55***							
Cr	−0.00	−0.03	0.03	0.14***	0.22***	0.30***	0.50***	0.45***						
MEA	0.20***	0.05	0.22***	0.26***	0.15***	0.48***	0.54***	0.41***	0.38***					
MMM	0.13***	0.07**	0.15***	0.28***	0.21***	0.49***	0.58***	0.52***	0.51***	0.69***				
IM	0.15***	0.07*	0.17***	0.22***	0.08**	0.43***	0.43***	0.34***	0.37***	0.48***	0.45***			
EM	0.21***	0.15***	0.27***	0.28***	0.13***	0.34***	0.23***	0.26***	0.13***	0.32***	0.28***	0.39***		
SE	0.15***	0.03	0.22***	0.24***	0.11***	0.38***	0.41***	0.34***	0.40***	0.45***	0.45***	0.59***	0.40***	
TA	0.07*	0.09**	0.15***	0.19***	0.22***	0.18***	0.11***	0.18***	0.09**	0.12***	0.16***	0.04	0.29***	0.07*

* p < 0.05, ** p < 0.01, *** p < 0.001, VF, Verification feedback; DF, Directive feedback; SF, Scaffolding feedback; TP, Teachers’ praise; TC, Teachers’ criticism; Re, Rehearsal; EI, Elaboration; Or, Organization; Cr, Critical thinking; MEA, Meta-cognitive awareness; MMM, Meta-cognitive monitoring and management; IM, Intrinsic motivation; EM, Extrinsic motivation; SE, Self-efficacy; TA, Test anxiety.

Before further exploring the relationships between teacher feedback and SRL among different grade levels, multi-group comparisons were conducted to test differences in such relationships across grades. First, a three-group SEM model as the baseline model, in which all the correlations were left free to vary by grade level and fit the data adequately (χ^2^ = 5976.432, *df* = 4,162, *p* < 0.001, RMSEA = 0.044, 90% CI [0.035, 0.050], CFI = 0.923, TLI = 0.916). Second, a constrained three-group SEM model was conducted, in which all the correlations were set to be equal across grades and fit the data adequately (χ^2^ = 6,847.356, *df* = 4,235, *p* < 0.001, RMSEA = 0.059, 90% CI [0.050, 0.067], CFI = 0.902, TLI = 0.905). Third, χ^2^-difference tests indicated that the model fit of the constrained model decreased compared with the unconstrained model [Δχ^2^_(73)_ = 870.924, *p* < 0.001], and other fit indices also became worse (ΔCFI > 0.02. ΔTLI > 0.01, ΔRMSEA > 0.01). This suggested that the constrained model was rejected and the relationship between teacher feedback and students’ SRL varied by grade level.

**FIGURE 3:**
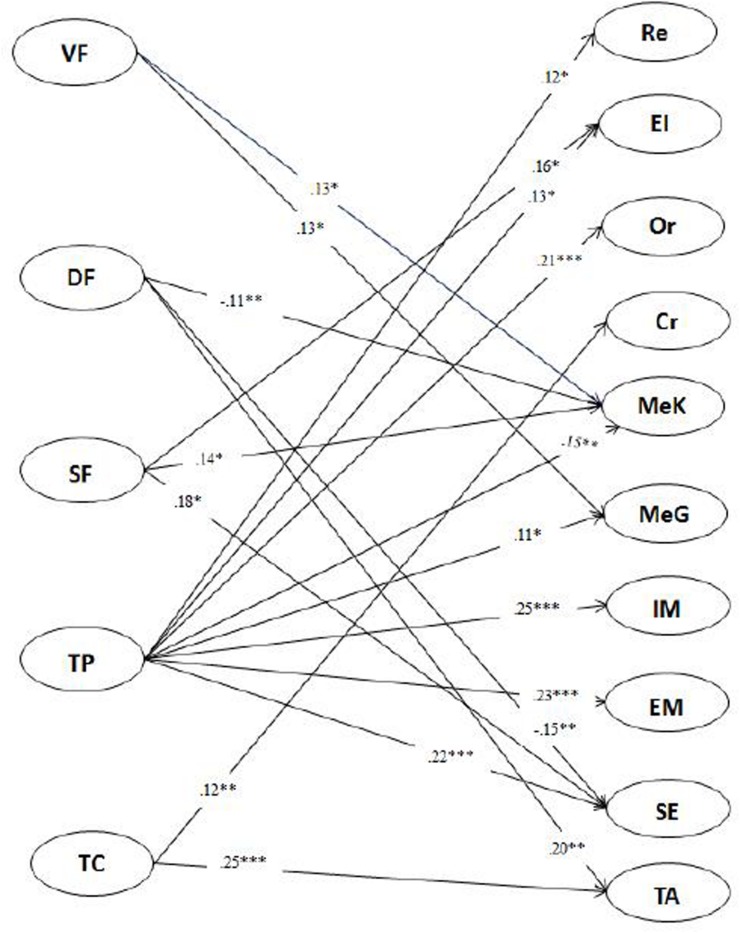
Relationship between 10th grade teachers’ feedback and students’ SRL.

As shown in Figure 3, for 10th-grade students, verification feedback had positive correlations with students’ metacognitive awareness and monitoring and management. Directive feedback had negative correlations with students’ metacognitive awareness and self-efficacy and was positively correlated with their test anxiety. Scaffolding feedback had positive correlations with students’ use of elaboration strategy, metacognitive awareness, and self-efficacy. Teachers’ praise had positive correlations with all components of students’ SRL, except the critical-thinking strategy use and test anxiety. The strongest correlation was with intrinsic motivation. Teachers’ criticism had positive correlations with students’ critical-thinking strategy use and their test anxiety.

**FIGURE 4:**
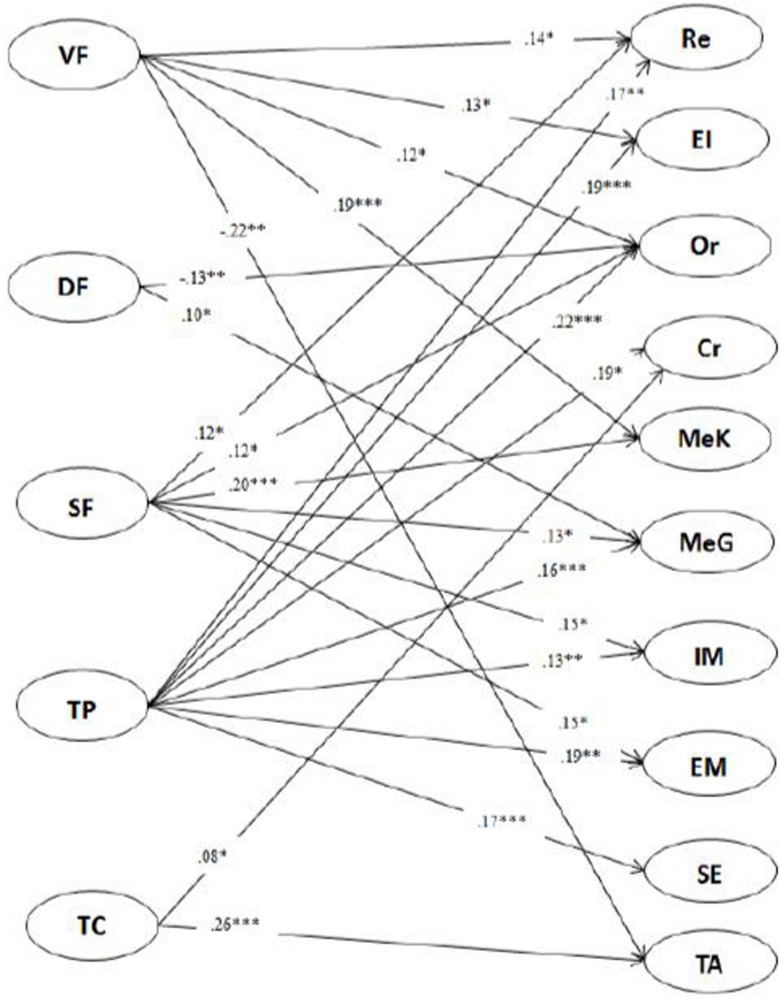
Relationship between 11th grade teachers’ feedback and students’ SRL.

As demonstrated in Figure 4, for 11th-grade students, verification feedback had positive correlations with students’ use of rehearsal, elaboration, organization strategies, and metacognitive awareness, and it had a negative correlation with their test anxiety. Directive feedback had a negative correlation with students’ use of organization strategy but a positive correlation with their metacognitive monitoring and management. Scaffolding feedback had positive correlations with students’ rehearsal and organization strategies, metacognitive awareness and monitoring and management, and intrinsic and extrinsic motivation. Teachers’ praise had positive correlations with students’ use of rehearsal, elaboration, organization, critical thinking, metacognitive monitoring and management strategies, intrinsic and extrinsic motivation, and self-efficacy. Teachers’ criticism had a positive correlation with students’ critical-thinking strategy use and test anxiety.

**FIGURE 5:**
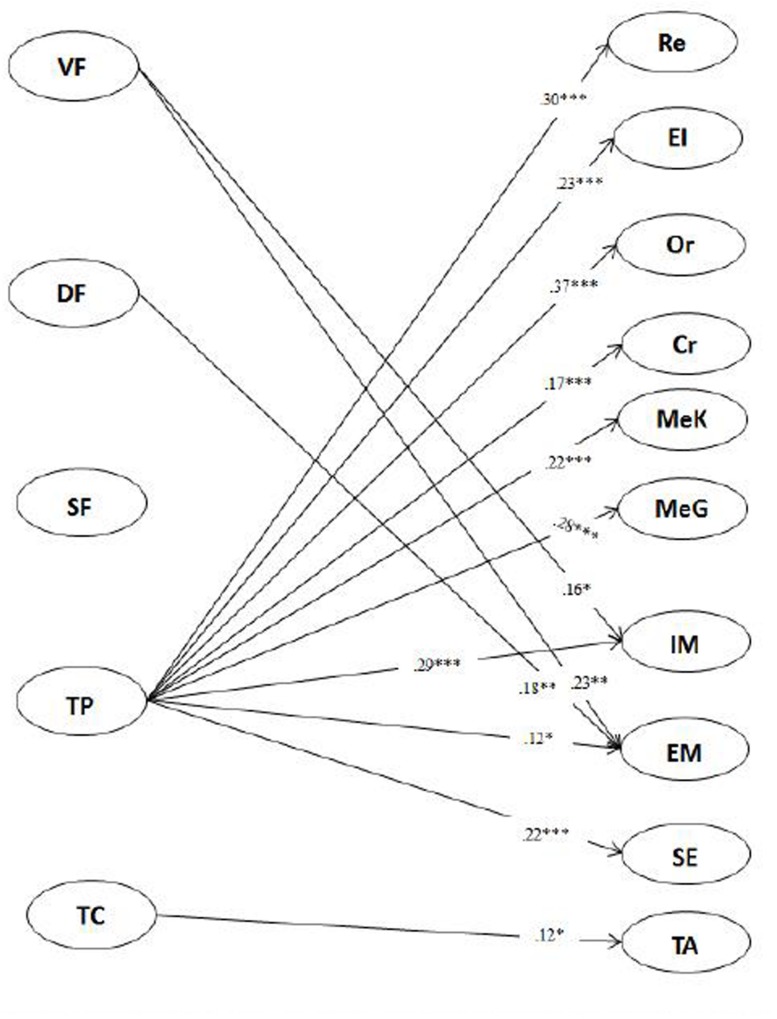
Relationship between 12th grade teachers’ feedback and students’ SRL.

As shown in Figure 5, for 12th-grade students, verification feedback had positive correlations with students’ intrinsic motivation and extrinsic motivation. Directive feedback only had a positive correlation with students’ extrinsic motivation. Scaffolding feedback had no significant correlations with students’ SRL. Teachers’ praise had positive correlations with all components of students’ SRL, except for test anxiety, with the strongest correlation being with their use of organization strategy (*r* = 0.37, *p* < 0.001). Lastly, teachers’ criticism had a positive correlation with students’ test anxiety.

## Discussion

### Grade-Level Differences in Teacher Feedback

The current findings generally supported our first hypothesis–that teachers were reported to provide feedback to students in different grade levels differently. Such differences may be linked to the learning phase students were in and teachers’ distinct goal structures across different grade levels (Cleary and Chen, 2009; Guo, 2017). However, the effect sizes of these grade-level differences were generally small, indicating that the grade level is not a decisive factor in explaining the differences.

First, students reported that 10th-grade teachers provided scaffolding feedback and praise with a higher frequency than did 11th- and 12th-grade teachers. This may suggest that 10th-grade teachers were perceived to be more patient in providing more scaffoldings, such as hints or clues, to facilitate students solving problems independently. In addition, it is also suggested that 10th-grade teachers were perceived to be inclined to be more positive and encouraging by providing more praise to students. This may be because 10th-grade teachers focused more on mastery- than performance-goal orientation. Their feedback may be more formative and significant for students in everyday learning (Graham, 2018; Hyland and Hyland, 2019). Their main goal was to help freshmen master certain knowledge and skills to prepare for future competitive entrance examinations since this is an important learning phase for developing good learning habits and abilities (Urdan and Midgley, 2003; Guo, 2017).

Second, 11th-grade teachers were reported to provide directive feedback with the least frequency as compared to the other-grade teachers. This suggests that 11th-grade teachers were reported to directly provide the correct answers or solutions to students. I posit that 11th graders are in a transitional phase–from freshmen to graduating students–and they may have been accustomed to the secondary school life and developed some learning habits; thus, they could proactively adjust their behaviors to meet teachers’ expectations (Guo, 2017).

Finally, 12th-grade teachers were reported to criticize students more as compared to 11th-grade teachers. This may be because the main goal of the 12th-grade level teachers was to help students prepare for the upcoming fierce competition they will face during entrance examinations (Hong et al., 2009). Thus, 12th-grade teachers were more likely to focus on performance-goal orientation and became stricter to push students forward as compared to their counterparts (Guo, 2017).

### Grade-Level Differences in Students’ SRL

The results generally supported previous research (Cleary and Chen, 2009; Hong et al., 2009; Lau, 2009; Yeung et al., 2011; Huang, 2013) and the second hypothesis that students’ SRL decreases with grade level. From an evolutionary perspective, this may be because, as students move to a higher grade, they gain an increased ability to evaluate their actual competence rather than being overly optimistic and exaggerated, such as when they were younger (Yeung et al., 2011). From a teaching perspective, this is probably because teachers in higher (vs. lower) grade levels emphasize competition more, which increases the pressure placed on students during the high-stakes public examinations held at the end of the 12th grade (Lee and Coniam, 2013; Lee et al., 2018). However, the effect sizes of these grade-level differences were generally small, suggesting that the grade level is not a determining factor in explaining students’ decline in SRL. In addition, as suggested by social cognitive theory (Bandura, 2011), students may be affected by their social environment, such as school climate. While adolescents are characterized by an increasing need for autonomy and self-consciousness, the environment of higher grades in high schools becomes more evaluative, competitive, and impersonal, which results in the progressive undermining of students’ SRL (Hong et al., 2009; Lau, 2009; Lee and Coniam, 2013).

### Grade-Level Differences in the Relationships Between Teacher Feedback and Students’ SRL

The findings indicated that teacher praise and criticism had similar relationships with students’ SRL, regardless of grade. Teacher praise was the most useful type of teacher feedback for all students to improve their SRL, regardless of grade. This can be explained by ERL theory–that praise, as an “external regulator,” can increase students’ positive proactivity and promote their active and adequate management of the regulation of their conduct and behavior (de la Fuente-Arias, 2017). This finding was in line with previous research that revealed that praise is a promoter of students’ strategy use and learning motivation (e.g., Harks et al., 2014; Guo and Wei, 2019; Hyland and Hyland, 2019), which consistently revealed the positive influence of teacher praise on student learning. In addition, teacher criticism, which may be considered as a “de-regulator” for students’ SRL (de la Fuente-Arias, 2017), was found to have limited negative correlations with students’ SRL, regardless of grade. This result was partly parallel to previous studies conducted in Western countries, which suggested the negative influence of criticism on students’ strategy use and learning motivation (e.g., Atlas et al., 2004; McMillan, 2014; Hyland and Hyland, 2019). Perhaps under Chinese Confucian Culture, which emphasizes harmony, self-control, and reserve (Tang, 2015), Chinese teachers tend to criticize students mildly to protect their egos, which is thus perceived by students as more acceptable and may not necessarily severely decrease their SRL (Guo, 2017).

Furthermore, the results also suggest that verification, directive, and scaffolding feedback had diverse relationship patterns with students’ SRL in language, which generally supported the third hypothesis. First, for 10th-grade students, verification feedback was only positively correlated with their use of metacognitive strategies, which was inconsistent with prior research findings that verification feedback, viewed as an “a-regulator” in student learning, played a negative role in students’ SRL (Lipnevich and Smith, 2008; de la Fuente-Arias, 2017; Guo and Wei, 2019). This may be because, as freshmen in senior high school, 10th-grade students tend to hold positive attitudes toward grades and could accordingly know their weaknesses and thus manage their cognition to perform better (Guo, 2017; Zhu and Mok, 2018). In addition, directive feedback, which can also be viewed as “a-regulator” in student learning (de la Fuente-Arias, 2017), was found to have more negative correlations with the SRL of 10th-grade students than with other-grade students’ SRL. This suggests that freshmen may be more severely affected by directive feedback because they are more likely to develop teacher dependency (Shute, 2008).

Second, scaffolding and verification feedback had more positive correlations with 11th-grade students’ SRL than for other students. This suggests that, for 11th-grade students, the more scaffolding and verification feedback teachers provide, the more SRL is fostered. These findings echo the previous research that scaffolding feedback, considered as an “external regulator,” plays a significant role in promoting students’ SRL (Finn and Metcalfe, 2010; McMillan, 2014; de la Fuente-Arias, 2017; Guo and Wei, 2019; Hyland and Hyland, 2019). However, surprisingly, our findings were inconsistent with prior research that revealed that verification feedback had negative influences on student learning (Lipnevich and Smith, 2008). This may be because 11th-grade students are in a very special learning phase–they have gradually adapted to the senior learning environment and begun to prepare for the competitive college entrance examinations. In this phase, they may become more sensitive to the feedback they obtain, which may promote them to use more learning strategies (Guo, 2017). In addition, students in Chinese cultural contexts have a strong conviction that they should work hard to perform well on competitive examinations; thus, when they lag behind or obtain low grades, they tend to work harder and become more self-regulated to increase their scores (Hui et al., 2011; Lau and Ho, 2016).

Finally, for 12th-grade students, verification and directive feedback had few positive correlations with their motivation in language, which were in line with previous research reporting that most students perceived teacher-led feedback as positive and constructive (Harris et al., 2014). Unexpectedly, scaffolding feedback had no significant correlations with their SRL; it seems that graduating students were less affected by these types of feedback. Owing to the upcoming competition of entrance exams in China, perhaps most students are less affected by external feedback. Furthermore, their learning focus may shift from mastery goals to performance goals, which may partly explain why scaffolding feedback–focusing on mastery goals–had no influence on their SRL. Whereas, notably, results indicated that teacher praise had stronger relationships with students’ use of rehearsal and organization strategies than with other SRL components. This suggests that, when praised by teachers, students were more apt to focus on their main learning goal, i.e., to practice, revise, or organize the testing of the material taught intensively in the previous 2 years to meet the upcoming *Gao Kao*. This may be because 12th-grade teachers tend to use praise to motivate students to achieve their learning goals (Guo, 2017).

## Implications

From a theoretical perspective, the current findings contribute to the body of research on teacher feedback and SRL and may inspire future research to take a differentiated perspective in the examination of them. I suggest that future research focuses on the grade-level differences of teacher feedback and students’ SRL in language and their relationships, rather than perceiving them as general traits across grades. Being aware of how teacher feedback and students’ SRL may differ across grades is critical for educational researchers, front-line teachers, and school administrators to better identify and address the learning needs and problems of different grade students.

From a practical perspective, considering that the relationship pattern between teacher feedback and students’ SRL across grades may differ, our findings provide useful insights for teachers to promote different grade students’ SRL by providing differentiated feedback. First, given that teacher praise had the most positive correlations and criticism had very limited negative correlations with all students’ SRL, regardless of grade, teachers should try to praise students more to encourage their self-regulation in language learning (Harks et al., 2014; Guo and Wei, 2019); in addition, they could also criticize them appropriately to help them correct their mistakes (Guo, 2017).

Second, given that verification, directive, and scaffolding feedback had different relationship patterns with students’ SRL in language, teachers should offer differentiated feedback to students in different grades to better cultivate their self-regulation. Specifically, for 10th-grade students, teachers should try to provide less directive feedback to avoid their dependency on teachers at the beginning of senior high school (Shute, 2008; Guo, 2017) and provide more scaffolding feedback and sincere and specific praise to facilitate students’ SRL (Clark, 2012; Zhu and Mok, 2018). For 11th-grade students, teachers may provide more scaffolding feedback and verification feedback to increase their use of SRL strategies and motivation in language (Finn and Metcalfe, 2010; McMillan, 2014; Guo and Wei, 2019; Hyland and Hyland, 2019). For 12th-grade students, however, considering that their SRL were less affected by these types of feedback, teachers may decrease these feedback and try providing them with more autonomy and time for self-assessment or peer-assessment, which may promote their SRL (Harris et al., 2015; Panadero et al., 2016).

## Limitations and Future Directions

This study had several limitations. First, the representativeness of this study was limited because the author collected research data from only one secondary school in Shanghai, China, although a large sample of students in this school participated. Therefore, the findings and conclusions of this study should be interpreted with caution. Future research should recruit larger and more representative research samples to increase the generalizability of results. Second, the findings were based solely on participating students’ self-reports, which are susceptible to response bias since students may overestimate their SRL abilities (Yeung et al., 2011). It is necessary for future studies to employ different research methods to provide stronger support for the findings. Third, this study focused only on the language curriculum; the grade-level differences in teacher feedback and SRL in other academic subjects remain unexamined. Finally, there was a lack of contextual data for further in-depth analysis. Future research focusing on a similar topic should collect more contextual data and strengthen the findings with more contextualization.

## Conclusion

This study confirmed our expectation that language teachers were perceived to provide feedback to students in different senior high school grades differently. Students reported that their SRL in language generally decreased with grade level. More importantly, regarding the relationships between different types of teacher feedback and students’ SRL, findings indicated that there were similar as well as different relationship patterns among different grade levels. Meanwhile, teacher feedback had a generally small to moderate impact on students’ SRL, regardless of grade. The representativeness of this study was limited. Therefore, the findings and conclusions of this study should be interpreted with caution. However, this study provides useful insight into teacher feedback and students’ SRL in different secondary grade levels in the Chinese-language context and deepens our understanding of teacher feedback and students’ SRL. The findings have significant psychoeducational implications for school policymakers, administrators, language teachers, and educational researchers in similar cultural contexts. Despite the limitations, this study adds new knowledge to the literature since multi-group SEM analyses helped to disclose grade-level differences in the relationships between different types of teacher feedback and students’ SRL in language education.

## Data Availability Statement

The datasets generated for this study will not be made publicly available because this data belongs to several people and is confidential. Should you have any enquiries, please direct them to: juan.wenguo@163.com.

## Ethics Statement

The studies involving human participants were reviewed and approved by The Chinese University of Hong Kong, Shatin, N. T., Hong Kong, China. Written informed consent to participate in this study was provided by the participants’ legal guardian/next of kin.

## Author Contributions

The author confirms being the sole contributor of this work and has approved it for publication.

## Conflict of Interest

The author declares that the research was conducted in the absence of any commercial or financial relationships that could be construed as a potential conflict of interest.
